# Electrochemotherapy in Aggressive Hemangioma of the Spine: A Case Series and Narrative Literature Review

**DOI:** 10.3390/jcm13051239

**Published:** 2024-02-22

**Authors:** Giuseppe Tedesco, Luigi Emanuele Noli, Cristiana Griffoni, Riccardo Ghermandi, Giancarlo Facchini, Giuliano Peta, Nicolas Papalexis, Emanuela Asunis, Stefano Pasini, Alessandro Gasbarrini

**Affiliations:** 1 Department of Spine Surgery, IRCCS Istituto Ortopedico Rizzoli, 40136 Bologna, Italy; giuseppe.tedesco@ior.it (G.T.); cristiana.griffoni@ior.it (C.G.); emanuela.asunis@ior.it (E.A.); stefano.pasini@ior.it (S.P.); alessandro.gasbarrini@ior.it (A.G.); 2 Department of Neurosurgery, IRCCS Istituto delle Scienze Neurologiche Bologna, Bellaria Hospital, 40139 Bologna, Italy; luigiemanuele.noli@ausl.bo.it; 3 Diagnostic and Interventional Radiology Unit, IRCCS Istituto Ortopedico Rizzoli, 40136 Bologna, Italy; giancarlo.facchini@ior.it (G.F.); giuliano.peta@ior.it (G.P.); nicolas.papalexis@ior.it (N.P.)

**Keywords:** hemangioma, spine, electrochemotherapy, bleomycin

## Abstract

(1) **Background:** this case series and literature review aims to evaluate the efficacy and safety of electrochemotherapy in the management of aggressive spinal hemangiomas, presenting two distinct cases. (2) **Methods:** we present two cases of spinal aggressive hemangioma which were refractory to conventional treatments and underwent electrochemotherapy. Case 1 involves a 50-year-old female who presented with an aggressive spinal hemangioma of L1, who previously underwent various treatments including surgery, radio-chemotherapy, and arterial embolization. Case 2 describes a 16-year-old female with a T12 vertebral hemangioma, previously treated with surgery and stabilization, who faced limitations in treatment options due to her young age and the location of the hemangioma. (3) **Results:** in Case 1, electrochemotherapy with bleomycin was administered following the failure of previous treatments and resulted in the reduction of the lesion size and improvement in clinical symptoms. In Case 2, electrochemotherapy was chosen due to the risks associated with other treatments and was completed without any adverse events. Both cases demonstrated the potential of electrochemotherapy as a viable treatment option for spinal hemangiomas, especially in complex or recurrent cases. (4) **Conclusions:** electrochemotherapy with bleomycin is a promising treatment for aggressive spinal hemangiomas when conventional therapies are not feasible or have failed. Further research is needed to establish definitive protocols and long-term outcomes of electrochemotherapy in spinal hemangioma management.

## 1. Introduction

An hemangioma is a benign tumor of the vasculature that develops from different blood vessel types [1]. In bone hemangioma, the proliferation of blood vessel cells happens within the bone tissue. Apart from hemangiomas of soft tissue, there is no consensus on the nature of these bone lesions, often being considered as vascular malformations rather than true neoplasms [2], and the term “hemangioma” has often been used in a generic and indiscriminate manner, resulting in the inclusion of lesions with different biology [3].

Vertebral hemangiomas (VHs) are extremely common lesions, with an incidence of 11% in the adult population. VHs can be observed at any age, even in children, with most diagnosed in the fifth decade of life. A slight female preponderance has been observed, with a male-to-female ratio of 1:1.5 [4,5].

VHs are a frequent and often incidental finding on computed tomography (CT) and magnetic resonance (MR) imaging of the spine. Radiographic, CT, and MRI images are all able to show distinctive features of capillary and cavernous VHs, leading to a radiological diagnosis [4].

Most VHs are latent (Enneking Stage 1, st.1). They are intercompartmental lesions, with well-defined margins, that grow slowly and then stop and do not require specific treatment [6]; only 1% of VHs become active and symptomatic. Approximately 55% of these symptomatic VH cases are associated with pain alone [7]. Some VHs may become aggressive and extend into the spinal canal and/or the paravertebral space, leading to a neurologic deficit (Enneking Stage 3, st.3) [8].

Symptomatic VHs can be treated with conservative medical therapy, percutaneous techniques, radiotherapy, surgery, or a combination of these [4,9,10]. The common treatments of choice in VHs without neurologic manifestations are vertebroplasty, kyphoplasty, and/or selective arterial embolization (SAE), with good pain control results. Decompression surgery and stabilization are highly recommended in patients suffering from neurological deterioration and symptoms, with SAE often used preoperatively. Radiation alone or as an adjuvant treatment is often used [10,11].

**Figure 1:**
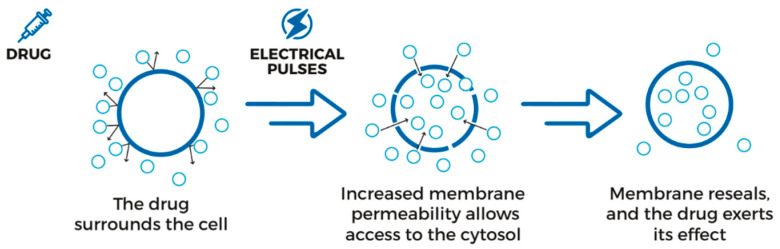
ECT mechanisms of action. The application of short- and high-intensity electric pulses allows a physically reversible phenomenon of increasing in cell membrane permeability, enhancing drug infusion in the tumor cells.

Electrochemotherapy (ECT) consists of a combination of electric pulses, led directly in the tumor in order to improve cell membrane permeability, and the infusion of chemotherapeutic drugs (Figure 1) [12]. Bleomycin and cisplatin have been shown to be the most effective and appropriate agents for electrochemotherapy in clinical use [13].

Bleomycin came out to be the best chemotherapy drug option for the treatment of hemangiomas in combination with electroporation. The risks and mechanism of cell toxicity are well described and known. Minor adverse reactions to bleomycin include nausea, vomiting, fever, and allergic reactions. Lung toxicity and pulmonary fibrosis represent by far the most serious adverse events related to bleomycin usage. Risk factors for bleomycin-induced lung damage include the following: age (over 70 years old), association with other chemotherapy drugs or radiation, dosage (with an incremented risk until 20% in case of more than 450 mg), and cumulative exposure [14]. Despite the potential threat, bleomycin is already one of the most frequently used sclerotherapy agents and ECT is safely applied in the treatment of vascular tumors [15]. Campanacci et al. [16] successfully used ECT and bleomycin infusion for the treatment of bone metastases and stressed how ECT-related drawbacks can be reduced with a proper preoperative planning and careful case selection.

Bleomycin has been widely used as an off-label treatment for various dermatologic indications. Gaudy et al., in 2006, first used ECT combined with intralesional bleomycin in 12 patients with skin melanoma, reporting improvement in local control and no systemic effects [17]. Recently, a systematic review assessed the safety and efficacy of the intralesional injection of bleomycin in skin hemangiomas, reported as a successful and well-tolerated treatment option, underlying a higher success rate when combined with electroporation in melanoma and warts [18].

Electroporation and ECT (with bleomycin) have been also used for the treatment of hepatic epithelioid hemangioendothelioma and hemangioma [19,20].

Evidence from the literature suggests the effectiveness of bleomycin in treating hemangiomas, with electroporation assuring the possibility to minimize drug dosage and related adverse events. The aim of this study is to present two cases of VHs treated with ECT with intravenous bleomycin infusion and summarize the current literature about alternative treatments for this lesion.

## 2. Materials and Methods

### 2.1. Patients

Given the novelty of this approach, the efficacy and safety of electrochemotherapy with bleomycin in patients with VHs are yet to be proven. For these reasons, only two patients were found eligible for such treatment and were included in this brief case series. Both patients had already received multiple treatments (including surgery) at other hospitals/health care providers. Other treatment options were also excluded due to plausible side-effects, drawbacks, and non-feasibility. Written consent for the treatment was obtained after a shared decision-making process with clinicians. Moreover, the patients signed the informed consent for a prospective observational study (registry) approved by the local Ethics Committee in December 2016 (protocol number 0022814).

### 2.2. Electrochemotherapy Procedure

According to guidelines and the literature, patients were interviewed for drug sensitivity and anesthesia-related issues; the drawbacks of bleomycin use in a specific case were evaluated accounting for the patient’s history and clinical documentation; ECG, hematic, and chemical exams were performed before treatment [16,21]. The entire procedure was carried out following the ESOPE guidelines [21]. The ECT mechanism of action is represented in Figure 1.

The electroporation system used was the Cliniporator^®^ VITAE (IGEA S.p.A., Carpi, Italy), already used in our institution for metastatic bone cancer treatment [16,22]. Hardware included trocar-type electrodes of diameter 1.8 mm. Electrodes used for both patients had a total length between 12 and 20 cm and were composed of an insulated protection part for soft tissue and an active non-insulated part of 3 cm. In cases number 1 and 2 reported here, 6 and 4 needles were used, respectively, to perform electroporation. In both cases, 15 mg/m^2^ of body surface of bleomycin were infused, following standard operating procedure [23] and according to a protocol successfully tested by Campanacci et al. [16] for the treatment of bone metastases.

To minimize possible side effects of this systematic-in-localized-fashion treatment, the two patients involved were not considered at risk for lung damage. Dosage-related risk was reduced thanks to the lower amount of bleomycin infused in a single, one-shot session compared to non-ECT drug infusion. According to protocols, FiO_2_ and oxygen flow were monitored during the procedure in order to reduce lung toxicity [23].

## 3. Results

### 3.1. Case 1

The first patient selected for electrochemotherapy was a female, 50 years old, with a history of multiple recurrent vertebral hemangioma in L1 previously treated at another institution. The patient’s medical history reported multiple skin cancers (ankle and thorax melanoma, both treated with superficial excision) and papillary thyroid cancer (treated with total thyroidectomy, leading to clinical hypothyroidism).

She was first diagnosed with L1 vertebral hemangioma of the bone in 2009 and consequently treated with en bloc resection and stabilization of T11-L3 due to neurologic impairment. In 2011, a CT scan detected the first, non-symptomatic, local recurrence, treated with 20 fractions of tomotherapy (40 Gy). Then, the lesion remained unchanged in size and symptoms for ten years. In June 2021, both MRI and CT scans detected a disease progression in the posterior area of T12 and L2 vertebral bodies. The patient was not experiencing any neurological impairment, but imaging clearly showed muscular (psoas) and neurological (spinal canal) involvement, leading clinicians to approach the local recurrence with SAE followed by five infusions of Denosumab in 3 months.

Despite the fact that an initial ossification process of the lesion was observable, a new MRI in April 2022 showed a slight increment of the lesion in the spinal canal, requiring another cycle of Denosumab. After a few months, in September 2022, the patient reported an almost sudden loss of lower limbs motor function and sphincteric control. An urgent intralesional decompression with pre-operative embolization was performed.

**Figure 2:**
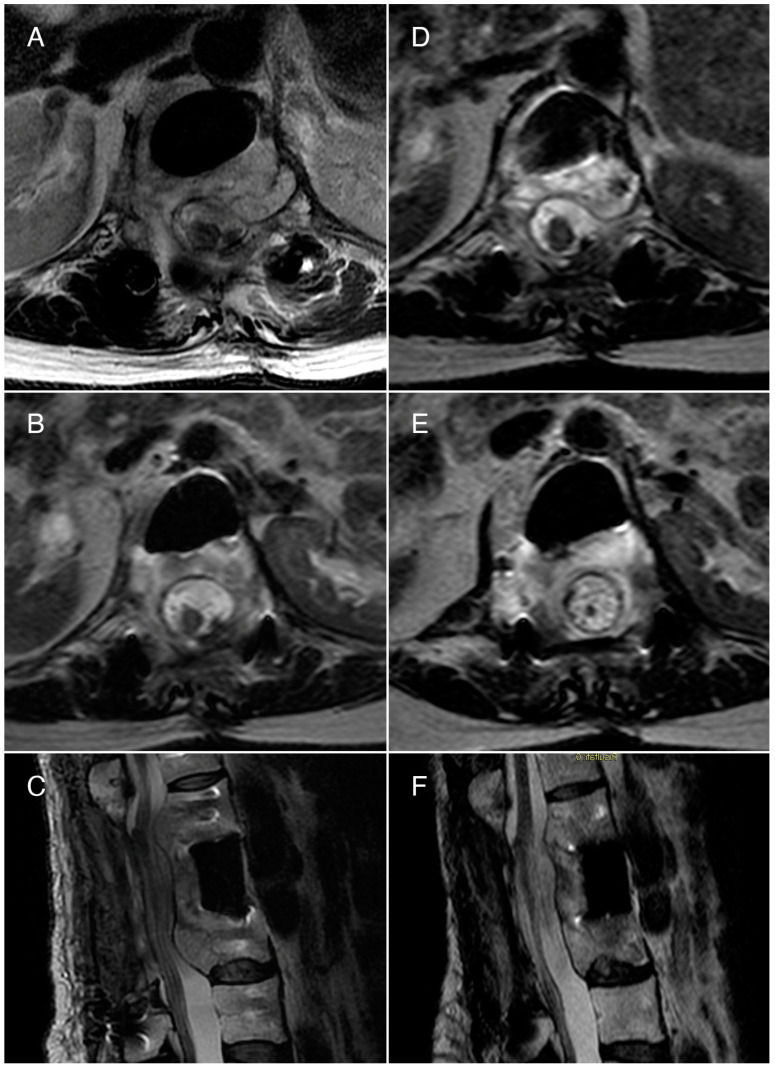
Case 1: T2-weighted MRI scans. (**A**,**B**) Axial views and (**C**) sagittal view performed before the first ECT treatment. The axial view at the L2 level shows a hemangioma causing compression of the dura and the cauda. The sagittal view reveals the hemangioma’s extensive invasion into the epidural space, both above and below the surgically removed L1 vertebral body, at the levels of T12 and L2. (**D**,**E**) Axial views and (**F**) sagittal view performed 12 months after the first ECT show a reduction of the compression caused by the hemangioma.

Due to the multiple recurrences, significant neurological symptoms, and the failure of various treatments, our team decided to enroll the patient in an electrochemotherapy trial. She received two treatments of electrochemotherapy with bleomycin at T12, L1, and L2 over four months, with no adverse events. The electrochemotherapy was performed by positioning six parallel needles adjacent to the vertebral bodies (two for each level) following the standard operating protocol for ECT as previously described [16,23,24]. For this patient, due to the presence of hardware and prothesis, a longer procedure time (a total time of 2 h and 45 min) and general anesthesia were required. The patient was intubated with low flow oxygen (<2 L/min) to reduce the risks of lung damage; before 15 mg/m^2^ of bleomycin were infused (2–3 min), clinicians made sure the FiO_2_ was under 30%. Eight minutes after infusion, pulses were applied. Two months after the initial treatment, MRI showed no significant changes compared to pre-electrochemotherapy. The treatment was repeated after four months with the same modalities, and a reduction in the lesion’s size was noted seven months after the second ECT, particularly in its intracanal component (Figure 2).

The radiological results were associated with improved clinical outcomes, in particular pain reduction, and no adverse events were reported.

### 3.2. Case 2

The second case eligible for electrochemotherapy was a 16-year-old female coming to our hospital from Greece. She was first diagnosed when she was 11 years old, in 2018. After months of experiencing nonspecific back pain (without neurological impairment), MRI and CT-guided biopsy confirmed the diagnosis of T12 vertebral hemangioma. Pre-operative SAE and intralesional excision were performed at another institution in Greece to achieve a local control of the disease; T10–L2 posterior fixation with T11–T12 interbody fusion was applied.

In 2022, a new MRI showed an increase in the lesion’s size compared to the previous year’s assessment. Both radiation (Cyber knife) and Denosumab treatment were considered too risky due to the posterior fixation construct and the young age of the patient, respectively.

In 2023, the patient came to our hospital, where a new CT-guided biopsy confirmed the diagnosis, and in July 2023, electrochemotherapy with bleomycin was performed. For this patient, the procedure took place without general anesthesia (no intubation or oxygen required) using deep sedation in spontaneous breathing; 15 mg/m^2^ of bleomycin were infused (2–3 min); 8 min later, pulses were applied. The procedure took 1 h and 15 min with no adverse event related to the treatment.

**Figure 3:**
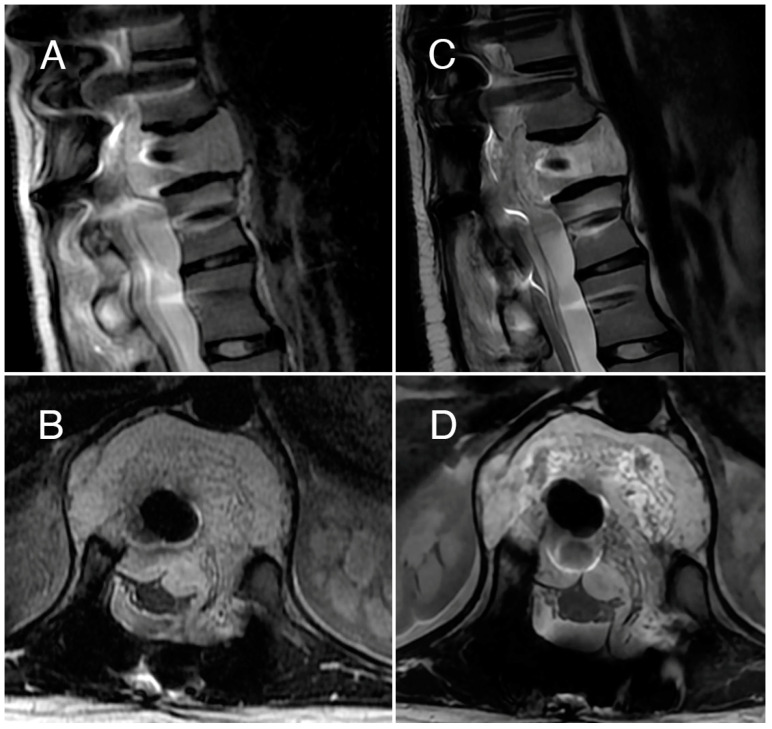
Case 2: T2-weighted MRI scans performed before (**A**,**B**) and 7 months after the ECT treatment (**C**,**D**). Imaging from before the treatment indicates a significant spinal cord compression at T12 level, which appears to be reduced after ECT treatment.

In an MRI performed two months after the treatment, the lesion appears to be stable (Figure 3) without a size reduction, and back pain is still occasionally present, even if no daily use of painkillers is necessary. The ECT treatment will be repeated to achieve a better improvement.

## 4. Discussion

*State of the art in VHs treatment*

Despite being a benign lesion and in most cases non- or mildly symptomatic, VHs could require treatment when the integrity/mechanical capacity of the bone or neurological structures are at risk or whenever symptoms have a significant impact on patients’ quality of life. Usually, typical and atypical VHs do not require treatment [4]. Laredo et al. [25] proposed a scoring system to describe the characteristics and progression of aggressive HVs, including the involvement of the vertebral body and posterior arch and cortical and soft tissue mass expansion. For these, several options are available depending on the type of hemangioma, position, and activity grade. Algorithms have been proposed, but still, there is no unanimous consensus on the best strategy [4,26,27].

*Radiofrequency ablation (RFA)*

Minimally invasive percutaneous techniques such as radiofrequencies/thermal ablation are widely used to treat benign tumors of the spine. RFA applies thermal energy through a generator used to deliver high-frequency alternating current, producing frictional heat and the ablation of tissues [1,28]. Encouraging data come from Tomasian et al. [29] who successfully treated two VHs patients without focal neurologic deficit, but further evidence is needed.

*Ethanol injection*

The intralesional injection of ethanol allows local vascular thrombosis and the destruction of the endothelium, resulting in devascularization, shrinkage of the lesion, and relief from pain and neural compression symptoms [30]. In 2017, Premat et al. [31] evaluated the efficacy and safety of obtained embolization via ethanol injection combined with percutaneous vertebroplasty with mixed results. Severe drawbacks, such as osteonecrosis, spinal cord ischemia, and vertebral collapse, have been reported following the ethanol injection procedure [30,32,33,34]. The introduction of more effective and safer treatments made ethanol injection fall out of favor.

*Radiation therapy*

Radiotherapy is often successfully used as a standalone practice in pain reduction for patients with VHs whose neurologic symptoms seem to be stable or slowly developing [35]. The target tissue is the abnormal vasculature within the hemangioma. Radiotherapy causes circulation to reduce and the consequent reduction in the lesion’s size. A dose-effect relation is proven to exist, with 30–40 Gy/25 fractions widely considered as the gold standard to reduce pain and achieve local control of disease [36]. It remains controversial to use radiotherapy as a stand-alone to treat VHs because of the delayed symptom relief [4]. In fact, it seems to not have a radiographically demonstrable effect on the surrounding bone tissue, making it not as useful for patients in which compression is mostly caused by focal bone hypertrophy [37]. Therefore, radiation is nowadays often used as an adjuvant technique after surgery to achieve better control of disease. In cases of incomplete tumor excision, recurrence and consequent exacerbating symptoms may occur with a 30–50% rate [1].

*Embolization*

Endovascular embolization is one of the most used and safest procedures to put in place once VHs are diagnosed. Besides being considered mostly as a neo-adjuvant procedure to reduce the risk of bleeding during surgery [38], it has been used as a definitive treatment as well [37]. Not all patients responded as expected to embolization only, with several studies showing no significant differences in patients’ symptoms and imaging after treatment [39].

*Surgery*

Rapid neurological deterioration represents the main indication for surgery, along with clear radiographic signs of instability. In order to avoid further progression and damage to neural structures (both nerve roots and spinal cord), a wide spectrum of surgery choices can be considered, from minimally invasive decompression as a standalone procedure, to decompression and stabilization, to vertebrectomy and consequent reconstruction [4]. As mentioned above, other adjuvant (such as radiotherapy) or neo-adjuvant (i.e., embolization) treatments can be administered to maximize therapeutic effects or minimize intra-operative risks [38].

Vertebroplasty is still considered an option for neurologically stable patients who suffer from compression fractures or large soma distortion to provide quick relief from pain [1]. Excluding the well-known risks of this augmentation procedure (i.e., cement leakage), vertebroplasty is not widely used since a recurrence could make symptoms of neural compression even worse. Results for vertebroplasty used as a standalone procedure to obtain a mechanical hemostatic embolization in aggressive VHs (with cortical/epidural involvement) are inconsistent [10,26,37].

*Treatment rationale for ECT*

As mentioned, both patients included in this case series had already received multiple treatments which were ineffective in the prevention of local recurrence, progression of disease, and symptoms’ control. Some drawbacks made clinicians exclude other treatment approaches. For the second case, radiotherapy was excluded due to the presence of instrumentation; denosumab was not considered feasible and safe for a young woman, and its effectiveness is yet to be proven. Further surgeries would have exposed both patients to the risk of potentially severe adverse events without giving them any certainty to prevent new recurrence and consequent neurological symptoms.

Surgery faces several risks, both intra- and postoperatively, for patients with VHs and should remain the last approach when neurological integrity is in rapid degradation. Embolization seems to be more effective when combined with surgery. Radiotherapy, ablation techniques, and ethanol injections are not proven to be effective as standalone treatments. Radiotherapy is useful in patients who have undergone incomplete excision of the lesion. All these non-surgical approaches are limited in their use due to a lack of definitive evidence. Despite being considered minimally invasive, thermal ablation and ethanol injection cause an irreversible alteration of cells and bone structure that must be taken into account, especially considering the benign nature of this lesion.

Electroporation uses short intense electrical pulses to increase temporarily the permeability of pores in the cell membrane, without compromising cells’ viability and allowing the transmembrane transportation of molecules and drugs [22]. A larger amount and bigger molecules can be delivered into the targeted cells and, for bleomycin, studies have shown a cytotoxicity increased by 300–500 times [40,41]. Electroporation seems to be also able to modify the blood supply in the tumor tissue, acting as an anti-vascular agent for cancer cells, providing, on the other hand, a rapid restoration of blood supply to healthy tissues [42].

Gasbarrini et al., in 2015, first published a preliminary report on the usage of ECT with bleomycin in patients with metastatic spinal melanoma, with promising results in terms of local control, symptom management, and safety. No serious adverse events related to the electric field applied and no bleomycin toxicity were reported for bone and surrounding tissues [43]. ECT with bleomycin then came out to be a valuable tool in pain reduction and improving the quality of life in patients affected by other bone metastases [16,44] and vascular malformation [15]. Osteolytic vertebral metastases may present with cortical expansion, soft tissue masses, and vertebral compression fractures, mimicking aggressive VHs [22]. Tschon et al. conducted a preclinical study with the aim to evaluate the effects of ECT on bone tissues and other relevant spine structures [45]. In fact, in contrast with other ablative techniques, electroporation seems to not interfere with healthy bone mineral structure and does not prevent osteogenic activity [16,46]. ECT, due to its non-thermal properties, maintains the extracellular matrix structure, with clear evidence of newly formed bone (osteoblast and osteoclast spread, oriented in the direction of lamellae contained in bone lacunae) in the preexisting trabeculae surrounding the electrodes [45]. Even irreversible electroporation (cleared as a non-thermal soft tissue ablation modality, leading cells to apoptosis) obtained with higher electric fields applied, which may partially occur during reversible ECT procedures, has been proven to not interfere with bone structure. In addition, there is emerging evidence that apoptotic cell death caused by electroporation even promotes bone growth and the renewal of bone structure [47].

ECT, despite focusing drug’s effects locally, cannot be considered as a minimally invasive procedure. The intravenous infusion of bleomycin (even if at a lower dosage) implies risks that require a meticulous operating plan and a careful patient-centered assessment of risk–benefit ratio.

## 5. Conclusions

Our preliminary results suggest that ECT with bleomycin can be considered an alternative treatment for patients with VHs that unsuccessfully underwent other treatments. No adverse events were registered during the procedure and at follow-up visits, even if the small number of patients and short follow up cannot guarantee the safety of the procedure and its results. Imaging for the first patient in this case series showed a decrease in the lesion size, in particular for its intracanal component, with no signs of damage in healthy adjacent tissues. It should be stressed that radiological results, which may require more than one treatment to be shown, should not be considered as the only endpoint to detect response or as the solo outcome measurement. Patients’ symptoms, histology signs of apoptosis, and reduced vital tissue are more appropriate to evaluate the response to treatment [48].

Due to its precisely targeted effect and its capacity to preserve tissues’ integrity and structural functionality, ECT may represent a turning point in the treatment of VHs. Further, ECT seems to maintain its safety when repeated, making it an essential tool in the case of recurrence, especially when conventional treatments fail or are not feasible.

A larger number of patients and longer follow-up periods are necessary to confirm these encouraging results and prove long-term effectiveness on local control and recurrence prevention.

## Data Availability

Research data are stored in the digital repository of Istituto Ortopedico Rizzoli.
